# GFRA3 promoter methylation may be associated with decreased postoperative survival in gastric cancer

**DOI:** 10.1186/s12885-016-2247-8

**Published:** 2016-03-16

**Authors:** Lars Lohne Eftang, Jovana Klajic, Vessela N. Kristensen, Jörg Tost, Qin Ying Esbensen, Gustav Peter Blom, Ida Rashida Khan Bukholm, Geir Bukholm

**Affiliations:** Department of Clinical Molecular Biology and Laboratory Science (EpiGen), Akershus University Hospital, Division of Medicine, Lørenskog, Norway; Department of Gastrointestinal Surgery, Akershus University Hospital, N-1478 Nordbyhagen, Lørenskog, Norway; K.G. Jebsen Center for Breast Cancer Research, Institute for Clinical Medicine, Faculty of Medicine, University of Oslo, Oslo, Norway; Department of Genetics, Institute for Cancer Research, OUS Radiumhospitalet Montebello, Oslo, Norway; Laboratory for Epigenetics and Environment, Centre National de Génotypage, CEA – Institut de Génomique, Evry, France; Department of Pathology, Akershus University Hospital, Lørenskog, Norway; Institute of Clinical Medicine, Akershus University Hospital and University of Oslo, Lørenskog, Norway; Infection Control and Environmental Health, Norwegian Institute of Public Health, Oslo, Norway; Department of Chemistry, Biotechnology and Food Science, Norwegian University of Life Sciences, Ås, Norway

**Keywords:** Gastric cancer, DNA methylation, Gene expression, *GFRA3*, Survival, Prognosis

## Abstract

**Background:**

A large number of epigenetic alterations has been found to be implicated in the etiology of gastric cancer. We have studied the DNA methylation status of 27 500 gene promoter regions in 24 gastric adenocarcinomas from a Norwegian cohort, and aimed at identifying the hypermethylated regions. We have compared our findings to the gene expression in the same tissue, and linked our results to prognosis and survival.

**Methods:**

Biopsies from gastric adenocarcinomas and adjacent normal gastric mucosa were obtained from 24 patients following surgical resection of the tumor. Genome-wide DNA methylation profiling of the tumor and matched non-cancerous mucosa was performed. The results were compared to whole transcriptome cDNA microarray analysis of the same material.

**Results:**

Most of the gene promoter regions in both types of tissue showed a low degree of methylation, however there was a small, but significant hypermethylation of the tumors. Hierarchical clustering showed separate grouping of the tumor and normal tissue. Hypermethylation of the promoter region of the *GFRA3* gene showed a strong correlation to post-operative survival and several of the clinicopathological parameters, however no difference was found between the two main histological types of gastric cancer. There was only a modest correlation between the DNA methylation status and gene expression.

**Conclusions:**

The different DNA methylation clusters of the tumors and normal tissue indicate that aberrant DNA methylation is a distinct feature of gastric cancer, although there is little difference in the overall, and low, methylation levels between the two tissue types. The *GFRA3* promoter region showed marked hypermethylation in almost all tumors, and its correlation with survival and other clinicopathological parameters may have important prognostic significance.

**Electronic supplementary material:**

The online version of this article (doi:10.1186/s12885-016-2247-8) contains supplementary material, which is available to authorized users.

## Background

Gastric cancer (GC) is second to lung cancer in worldwide cancer-related deaths, and is the result of a complex interplay between chronic *Helicobacter pylori* infection, human genetic factors and environmental carcinogens. However, it is becoming increasingly clear that cancer development is as much a result of epigenetic alterations, as it is a genetic disease [1]. Genetic mutations are relatively infrequent in GC, whereas epigenetic alterations such as DNA methylation may be much more important in promoting GC [2]. DNA methylation is an heritable modification of gene activity, which does not make alterations to the DNA sequence, but involves attachment of a methyl group to the carbon 5 position of cytosines, most commonly where cytosine occurs next to guanine, separated by phosphate known as a CpG dinucleotide. Most focus has been directed at DNA hypermethylation, however whole genome hypomethylation is prevalent in several cancers [3–5]. Hypermethylation of gene promoter regions may result in silencing of tumor suppressor genes, however the role of hypomethylation has been less clear, but may be associated with increased expression of oncogenes [6]. A distinctive DNA methylation phenotype has been identified in colorectal cancer (CIMP) [7], and particular methylation patterns have been associated with subgroups of breast cancer [8], lung cancer [9] and glioma [10]. A CIMP in gastric cancer has been suggested, but is controversial [11]. Several causes of aberrant DNA methylation in GC have been established, including aging, dietary causes and microorganisms such as *H. pylori* and Ebstein-Barr virus [12–16].

In a previous study we compared the gene expression profile of 20 gastric tumors against matched non-cancerous mucosa. We identified the most differentially expressed genes and related these to postoperative survival [17]. Nine genes relevant to gastric carcinogenesis had previously shown similar expression patterns in *H. pylori* exposed gastric mucosa cells in vitro [18]. We suggested that the increased expression of these genes in the gastric tumors may represent early events in gastric carcinogenesis mediated by chronic *H. pylori* infection, in particular claudin-1 (*CLDN1*) and interleukin-8 (*IL-8)*.

The aim of the present study was to examine the DNA methylation status in gastric adenocarcinomas whose gene expression were previously determined by cDNA microarrays [17]. We aimed at assessing the overall state of hypo- or hypermethylation in the tumors, and identify whether there was an association between the DNA methylation status, clinicopathological factors and gene expression.

## Methods

### Tissue and patient characteristics

Patients with non-cardia gastric adenocarcinoma were identified during upper endoscopy at the outpatient clinic at Akershus University Hospital, Norway, with a histopathological diagnosis of diffuse or intestinal type GC. Thoraco-abdominal computed tomography imaging was performed to exclude patients with distant metastatic disease, ineligible for curative surgery. On admission for surgery, written, informed consent was obtained from all participants in the study. Immediately following the removal of the principal surgical specimen, two tissue samples from each patient were obtained: one from the tumor mass border and another from healthy gastric corpal mucosa, at least 5 cm away from the tumor. The samples were immediately fresh frozen on dry ice, before definitive storage at −80 ° C. All sample acquisition and handling were performed by the same individual. The tumors were verified and further classified by two senior specialist pathologists. The study was approved by the Regional Ethics Committee (REK) and the ethics committee at Akershus University Hospital. All samples and patient data were coded and blinded before analysis. Patients, clinicopathological characteristics, diagnostic are presented in Table 1.

**Table 1:** Patient characteristics and clinicopathological features of the 24 gastric tumors used in the study

Number and sex of patients		*n* = 24 (females *n* = 7, males *n* = 17)
Ethnicity		Caucasian *n* = 21 Asian *n* = 3
Age at surgery		Total: 68.4 years (±12.3)
		Females: 65.4 years (±21.5)
		Males: 69.5 years (±8.5)
Postoperative survival (deceased individuals)		13.3 months (±8.8)
Postoperative survival (alive individuals at study end)		46.0 months (±8.0)
Tumor size		47 mm (±28)
Tumor stage	T1	3
	T2	13
	T3	5
	T4	3
Nodal stage	N0	11
	N1	8
	N2	3
	N3	2
Histological type	Intestinal	6
	Diffuse	14
	Mixed	4

Values are the mean plus/minus standard deviation where appropriate. The details of gender associated with death and survival have been combined

### Methylation assays

Total DNA was extracted from the fresh frozen tissue using Dneasy Blood and tissue kit (Qiagen GmBH, Germany) according to the manufacturer’s standard preparation protocol. DNA concentration and 260/280 ratio were then assessed using a NanoDrop ND-1000 spectrophotometer (NanoDrop Technologies, USA), and found to be adequate for further analysis. 500 ng of DNA was bisulphite converted using the EpiTect 96 Bisulfite Kit (Qiagen GmbH, Germany). Effective bisulphite conversion was verified by absolute quantification assay using Applied Biosystem7900HT/7900HTFast Real Time PCR System Amplification and pairs of primers specific for either converted or unconverted DNA. An aliquot of 4 μL of the bisulphite converted DNA was used to perform genome-wide DNA methylation profiling of 24 gastric tumors against matched non-cancerous mucosa using the Illumina HumanMethylation27 BeadChip. This platform detects the methylation status of 27 578 different CpG sites in >14 000 promoters in the human genome. The experiment was performed using the Infinium Methylation Assay Experienced User Card protocol. All steps were performed according to the Infinium protocol.

### Immunohistochemistry

The presence of *H. pylori* in the surgical specimens was analyzed using a polyclonal anti-Helicobacter-antibody (Dako, Denmark, code B0471, dilution 1:200). 4 μm sections of formalin-fixed, paraffin-embedded tissue from non-tumorous mucosa were applied on coated slides. Deparaffinization, rehydration and epitope retrieval were performed in a Dako PT Link (Dako, Denmark) at 97 °C for 20 min. The immunostaining procedure was carried out in a Dako Autostainer Plus applying the Envision™Flex, High pH system (Dako, Denmark).

### Statistical analysis

Data was processed using the *lumi* R package. All probes that contained a “zero” value in at least one sample for methylated and unmethylated signals were removed from further analysis. Intra-sample normalization consisted of color bias correction, which is the normalization between the two color channels, and background level correction, using the negative control probes present on the array. Lastly, quantile normalization was performed on the intensities of methylated and unmethylated probes separately, instead of the summarized methylation levels. β-values (the degree of methylation) were used for further analysis. The β-value at each CpG site represents continuous value from 0 to 1 where 0 is fully unmethylated and 1 is entirely methylated at that locus. The ∆β value is the difference between the β value of the tumor sample and that of the normal sample, ranging from −1 to 1.

The ∆β value was calculated for all CpG sites in all sample pairs, and the data were loaded into the J-express software package [19]. Rank product testing [20] was then performed to test whether the differential methylation between tumor tissue and matched normal mucosa was significant. The ∆β value was declared significant if the adjusted p-value, i.e. the FDR q-value, was less than 0.05. Hierarchical clustering was performed using utilizing the J-express software package [19].

The filtered dataset, consisting of the 200 most significant ∆β values, was imported into Pathway Express, part of the Onto-Tools software suite [21, 22], for KEGG (Kyoto Encyclopedia of Genes and Genomes) cellular signaling pathway analysis [23]. Pathway Express calculates an Impact Factor (IF), which is used to rank the affected signaling pathways, based on the fold change, the number of the involved genes in the pathway, and the amount of perturbation of downstream genes [24].

The filtered dataset was then entered into SPSS Statistics (IBM Software, New York, USA, version 22.0.0.1) to perform correlation analysis to select differentially methylated gene promoter regions that associated with gene expression and clinicopathological parameters. Both Pearson and Spearman correlation coefficients were employed to identify associations. In the one CpG site that was highly correlated to survival, different cut-off levels were applied to construct high and low methylated groups, before statistical significance between the groups was assessed using a log-rank (Mantel-Cox) test. A Kaplan-Meier survival plot was created to demonstrate the difference in survival between the high and low expression groups. Linear regression analysis was then performed, to predict determinants of *CLDN1* expression, using Stata (StataCorp LP, Texas, USA, version 13.1).

The DNA methylation data are available in the ArrayExpress database under the accession number E-MTAB-3813 (http://www.ebi.ac.uk/arrayexpress/experiments/E-MTAB-3813).

## Results

### General description

**Fig. 1:**
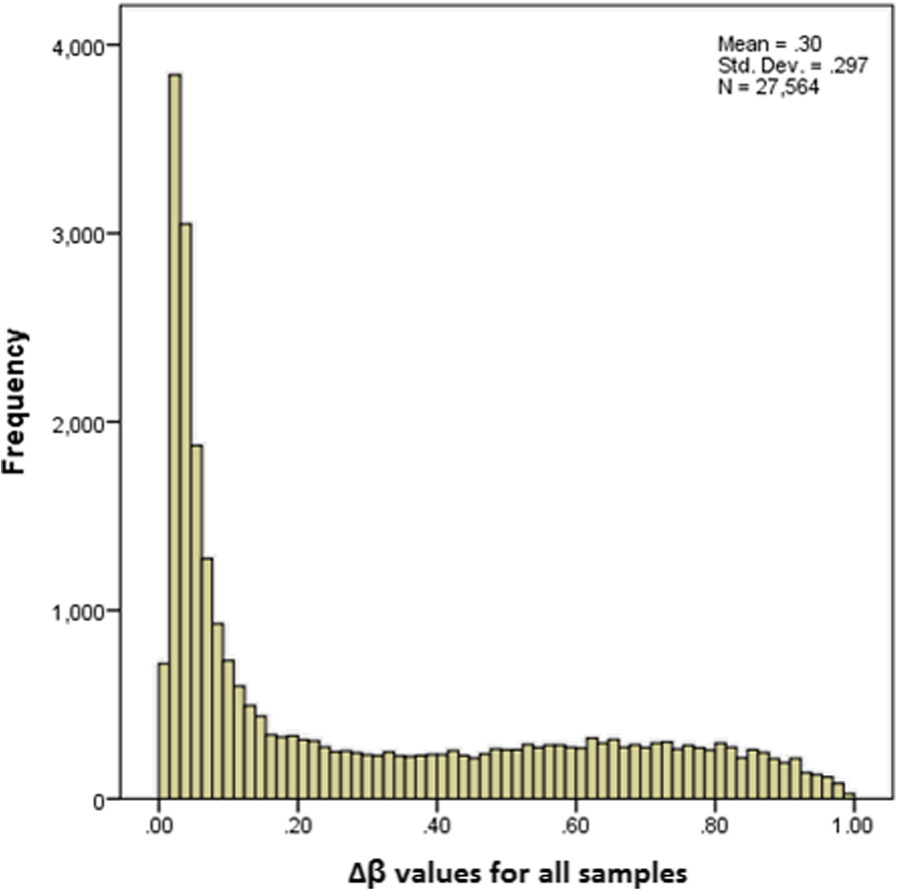
Histogram of the ∆β values. The frequency of the ∆β values in the dataset of >27 000 CpG sites show*s* that most CpG sites demonstrated little variance between gastric tumor and normal tissue

**Fig. 2:**
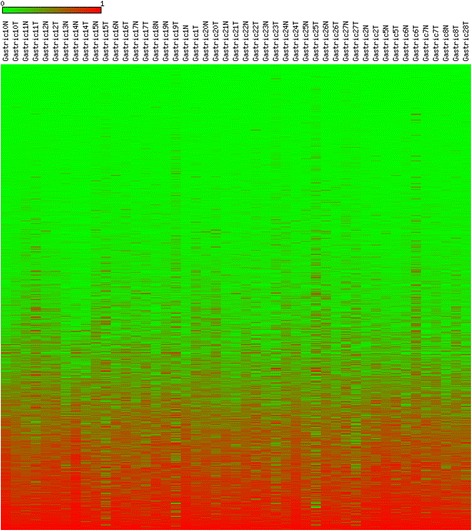
A heatmap presentation of the DNA methylation levels of the entire dataset of 27564 CpG sites in 24 tumor and normal gastric cancer tissue pairs. A value of 0.00 (most green) indicates fully unmethylated, whereas a value of 1.00 (most red) indicates entirely methylated locus. The figure demonstrates that the majority of CpG sites in the dataset, including both tumor and normal tissue, showed low levels of DNA methylation

To investigate the DNA methylation status of 24 GC samples and matched controls, more than 27 500 gene promoter sites were examined using the Illumina Infinium HumanMethylation27K platform. First, the methylation status of the entire dataset was considered. Most CpG sites, including both the tumor and normal tissue, demonstrated low levels of methylation, i.e. low β values. Second, there was little variance between the samples within each locus, even between the tumor and normal groups. This is illustrated in the histogram in Fig. 1 and in the colored heatmap in Fig. 2.

Then, the ∆β values for each CpG site were calculated. 53.1 % of all sites demonstrated a net positive value, whereas 46.9 % demonstrated a net negative value, indicating a slight overall increase in CpG methylation in the tumors.

### Rank product testing

Of the 27 500 CpG sites on the array, 1 660 CpG sites, corresponding to 1 194 genes, showed statistically significant increased methylation in the tumor relative to normal mucosa, whereas 1 276 CpG sites, corresponding to 1 017 genes, showed significant decreased methylation in the tumor relative to normal mucosa, supporting the trend from the entire dataset.

**Fig. 3:**
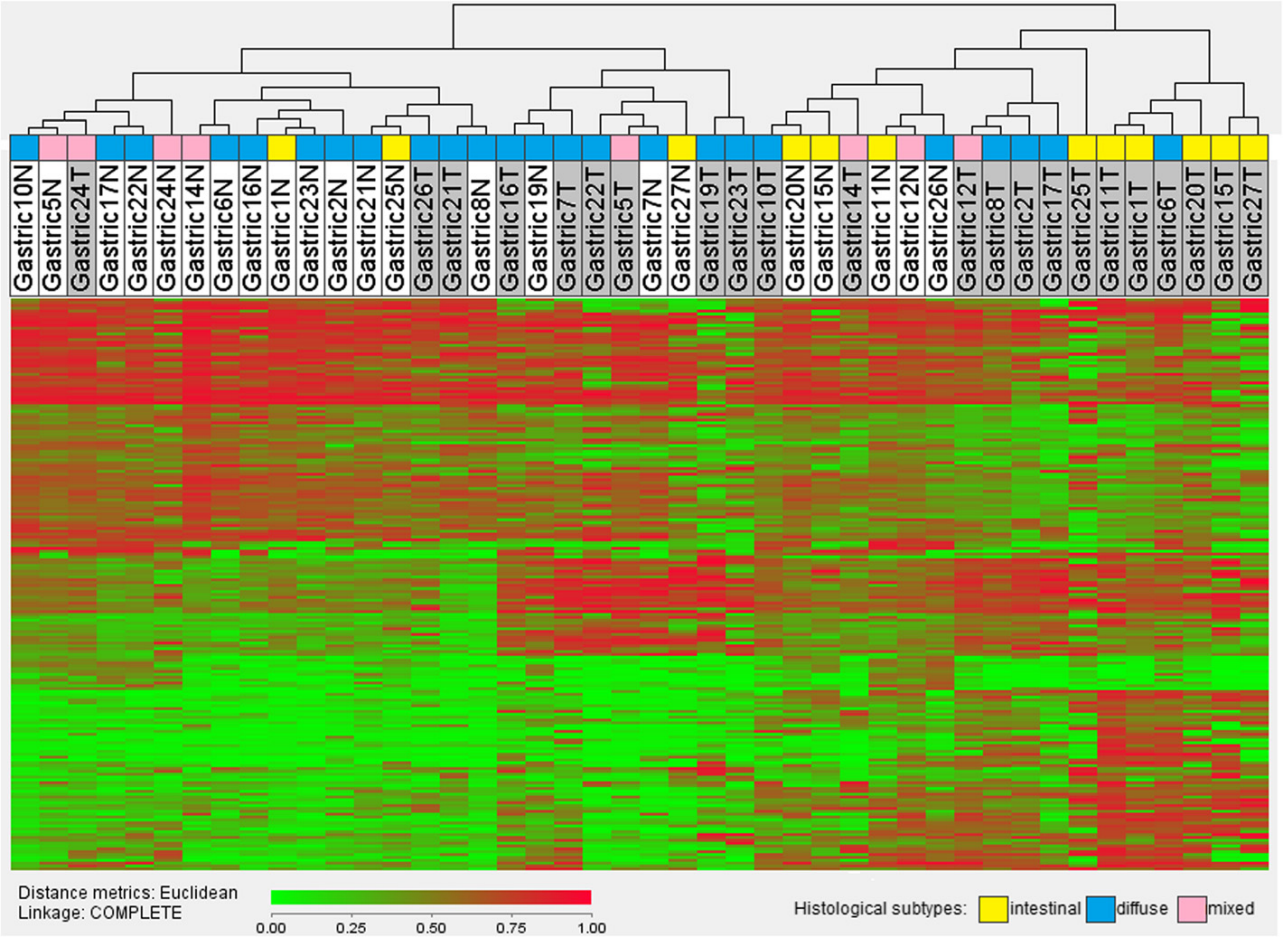
Hierarchical clustering of the normal and tumor samples. Top row: Tumor samples are shaded grey, normal gastric tissue samples are white. A value of 0.00 (most green) indicates fully unmethylated, whereas a value of 1.00 (most red) indicates entirely methylated locus. Most normal tissue samples seem to concentrate on the left side of the heatmap, whereas most tumor samples aggregate on the right, indicating similarities within the two groups

The dataset of all significant CpG sites was then subjected to hierarchical clustering using average linkage and Euclidean distance measure, using the J-express 2012 software package [19]. There was a tendency for the tumor samples to cluster together, and for the normal samples to cluster together, indicating stronger methylation similarities within each of the two groups, than within each of the sample pairs (Fig. 3).

### Filtering of statistical CpG sites

To produce a reasonably sized list of the most differentially methylated CpG sites, a list of the 100 most hypermethylated and the 100 most hypomethylated CpG sites in the tumor relative to the control were created, hereby referred to as the filtered dataset (Additional file 1).

### Clinicopathological correlation

The filtered dataset was then compared to post-operative survival, tumor size, Lauren classification, lymph node metastasis, gastric mucosa metaplasia and atrophy and *H. pylori*, listed in Table 2.

**Table 2:** Associations between hypermethylated and hypomethylated CpG sites and clinicopathological features

	Post-op survival	Lymph node metastasis	Tumor size	Lauren classification	Metaplasia	Atrophy	*H. pylori*
CpgID/Gene promoter Hypermethylated in the tumor							
cg09350274/GFRA3	*r* = −0.69 *p* = 0.002	*r* = 0.52 *p* = 0.028	*r* = 0.48 *p* = 0.42		*r* = 0.480 *p* = 0.044	*r* = −0.590 *p* = 0.01	
cg02720618/ESR1	*r* = 0.56 *p* = 0.016						
cg04623837/HCG9			*r* = 0.64 *p* = 0.004				
cg07307078/TUBB6			*r* = 0.49 *p* = 0.04				
cg08615333/TGFB3			*r* = 0.5 *p* = 0.037				*r* = 0.504 *p* = 0.033
cg01566170/CAPN2				*r* = −0.53 *p* = 0.023		*r* = 0.526 *p* = 0.025	
cg16986846/SCGB2A1				*r* = −0.576 *p* = 0.012			
cg02633817/FXYD3					*r* = −0.474 *p* = 0.047		
cg20640433/LAMA2					*r* = 524 *p* = 0.026		
cg21905630/GSH2					*r* = 0.52 *p* = 0.028		
cg13718960/RNASE1						*r* = 0.554 *p* = 0.017	
cg19118812/ELMO1						*r* = −0.546 *p* = 0.019	
cg26557658/FAM43B						*r* = −0.604 *p* = 0.008	
cg03616357/FLJ21159							*r* = 0.574 *p* = 0.013
cg08615333/TGFB3							*r* = 0.504 *p* = 0.033
cg14189571/ZFP42							*r* = 0.484 *p* = 0.042
cg17872757/FLI1							*r* = 0.495 *p* = 0.037
cg21790626/ZNF154							*r* = 0.573 *p* = 0.013
cg27546237/COL4A1							*r* = 0.527 *p* = 0.025
CpgID/Gene promoter Hypomethylated in the tumor							
cg13694749/SCN4A			*r* = −0.638 *p* = 0.004				
cg18059088/HS3ST1	*r* = −0.474 *p* = 0.047						
cg26619317/CNN3	*r* = 0.481 *p* = 0.043						
cg07131544/NCR2		*r* = 0.496 *p* = 0.036					
cg14696870/FCER1A				*r* = −0.567 *p* = 0.014			
cg20676475/LCE3D				*r* = −0.708 *p* = 0.001			
cg00974864/FCGR3B				*r* = −562 *p* = 0.015			
cg13180098/RHO				*r* = −0.604 *p* = 0.008	*r* = −0.597 *p* = 0.009	*r* = 0.682 *p* = 0.002	
cg24691453/S100A4				*r* = 0.470 *p* = 0.49			
cg15309006/LOC63928				*r* = −0.499 *p* = 0.035			
cg26789453/TMEM116				*r* = −0.512 *p* = 0.030			
cg26264314/NALP5				*r* = −0.474 *p* = 0.047			
cg20383064/BFSP2				*r* = −0.487 *p* = 0.040			
cg25119415/MNDA				*r* = −0.646 *p* = 0.004	*r* = −0.514 *p* = 0.029	*r* = 0.547 *p* = 0.019	
cg14603345/BTBD3					*p* = −0.483 *r* = 0.042		
cg17356733/IFNGR2						*r* = −0.480 *p* = 0.044	
cg23756272/BCL2						*r* = −0.500 *p* = 0.035	
cg00842351/TJP2						*r* = −527 *p* = 0.24	
cg25248094/SH2D1A						*r* = 0.487 *p* = 0.041	*r* = −0.526 *p* = 0.025
cg04454050/TREML1							0.474 0.047
cg02611419/KCNK17							*r* = −502 *p* = 0.034
cg05252264/FCAR							*r* = 0.587 *p* = 0.10
cg22268164/TRHR							*r* = −0.619 *p* = 0.006
cg02046017/LOC220070							*r* = 0.475 *p* = 0.046
cg09191232/PAPSS1							*r* = −0.581 *p* = 0.011

The filtered list of 100 hypermethylated and 100 hypomethylated CpG sites were compared to associated gene expression. 19 of the hypermethylated CpG sites and 25 of the hypomethylated CpG sites showed significant correlation with one or more of the clinicopathological parameters (Pearson correlation coefficients (r) and significance levels (p) are listed)

**Fig. 4:**
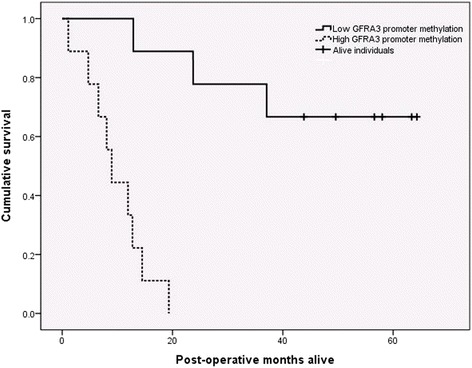
Kaplan Mayer survival plot of patients with resected gastric tumors. High and low methylated GFRA3 groups were constructed, using the mean value as the group divider. Individuals with hypermethylation of the GFRA3 promoter region showed a highly unfavorable prognosis, whereas individuals with a low degree of methylation at that locus demonstrated a relatively good prognosis

Although 44 CpG sites showed relationship with one or more of the factors, the promoter region of the *GFRA3* gene showed significant relationship with nearly all the clinicopathological factors. Firstly, an inverse relationship was detected between the *GFRA3* promoter ∆β values and post-operative survival (*p* = 0.01). High and low methylated *GFRA3* groups were constructed using the *GFRA3* promoter ∆β mean (*p* = 0.017) as the group divider, as demonstrated in the Kaplan-Meier survival plot in Fig. 4. Secondly, a strong, and highly statistically significant positive relationship was identified between *GFRA3* ∆β and gastric atrophy (*p* < 0.001). Thirdly, there were statistically significant, although slightly weaker positive correlations between *GFRA3,* ∆β and lymph node metastasis (*p* = 0.028), the degree of gastric mucosa metaplasia (0.044) and also patient age at surgery (*p* = 0.038). There was no association between the *GFRA3* promoter methylation levels and histological subtype or *H. pylori* status*,* nor did we find significant correlation to *GFRA3* gene expression extracted from our previous publication [17].

A multivariable Cox regression was then performed. Controlling for age at surgery, tumor size and histological type, *GFRA3* methylation level was still a highly significant (*p* = 0.01) predictor of survival.

### Gene expression correlation

The filtered dataset was compared to corresponding gene regulation, imported from our previous study [17], to analyze whether the gene promoter methylation status could account for aberrant gene expression. Of the 100 most hypermethylated CpG sites in the tumor relative to the control, four CpG sites showed a statistically significant inverse correlation with gene expression, listed in Table 3. Among the 100 most hypomethylated CpGs, six CpG sites showed significant inverse relationship with gene expression (Table 4).

**Table 3:** Correlation between hypermethylated gene promoters and corresponding gene expression

Gene promoter/gene	Pearson coefficient	*P* value	Spearman’s rho	*P*-value
CPD		NS	−0.482	0.043
DEFB106A	−0.531	0.23	−0.529	0.024
FCGR3B	−0.575	0.13	−0.569	0.014
GATA4		NS	−0.643	0.004

The 100 most significant hypermethylated gene promoter sites were correlated with their respective gene expression. Only statistically significant correlations are shown (*p* < 0.05)

**Table 4:** Correlation between hypomethylated gene promoters and corresponding gene expression

Gene promoter/gene	Pearson coefficient	*P* value	Spearman’s rho	*P*-value
DHX32	−0.495	0.037	−0.624	0.006
PPYR		NS	−0.490	0.039
FOXI1	−0.683	0.002	−0.511	0.03
ORM1	−0.594	0.009	−0.670	0.002
ZSCAN18		Ns	−0.511	0.030
PXDN	−0.480	0.44	−0.579	0.012

The 100 most significant hypomethylated gene promoter sites were correlated to their respective gene expression. Only statistically significant correlations are shown (*p* < 0.05)

The 130 most differentially regulated genes in the tumors [17] were then compared against DNA methylation (Table 5). Three genes showed an inverse relationship with the methylation status of their gene promoter region.

**Table 5:** Correlation between the most differentially regulated genes and their promotor methylation

Gene promoter/gene	Pearson coefficient	*P* value	Spearman’s rho	*P*-value
SPP1		ns	−0.523	0.026
ALDH3A1		ns	−0.560	0.016
TCN1		ns	−0.521	0.027

The 130 most differentially regulated genes imported from our previous paper [17] were compared to corresponding gene promoter methylation levels. Significant correlations are shown (*p* < 0.05)

**Table 6:** KEGG cellular signaling pathways

Pathway name	Impact factor	*p*-value
Natural killer cell mediated cytotoxicity	6.7	0.008
Hematopoietic cell lineage	5.1	0.017
Colorectal cancer	5.0	0.018
Cytokine-cytokine receptor interaction	5.4	0.022
Bladder cancer	4.8	0.029
Endometrial cancer	4.9	0.046

Significant associations between the 100 most hypomethylated CpG sites in the tumor relative to normal tissue, and KEGG cellular signal pathways (FDR corrected *p < 0*.05). The 100 most hypermethylated CpG sites did not significantly associate with any KEGG pathways

Then, to investigate the association between the most significant genes from our previous study [17], and GFRA3 gene methylation from the current study, linear step-wise regression was performed. *IL-8* gene expression (*p* = 0.006) and *GFRA3* methylation (*p* = 0.008) were highly associated with *CLDN1* expression.

### Cellular signaling pathways

Then the filtered dataset was analyzed for associated KEGG signal pathways using Pathway Express. Significantly impacted pathways and corresponding Impact Factor (IF) are presented in Table 6. None of the hypermethylated CpG sites (in the tumor relative to normal) associated with any KEGG pathways, while many of the hypomethylated CpG sites significantly associated with six cellular signaling pathways; including three cancer-related pathways.

### Assorted genes

Finally, the β and ∆β values for particular gene promoter regions were noted: *TIMP3*, *SEMA3B*, *FBP2*, *TEAD4*, *CDH1*, *CDKN2A*, *LOX*, *MLH1*, and *SFRP1, 2 and 5* (Additional file 1), which are addressed in the discussion.

## Discussion

### General description

Although there is currently a major interest in the relationship between DNA methylation and various human diseases, the significance of DNA methylation was highlighted more than 30 years ago by Ehrlich et al. [25], who showed that the extent of methylation varies significantly across human tissues. However, gastric tissue was not studied in detail. In this study we have characterized the DNA methylation pattern in 24 GCs compared to matched normal mucosa. Overall, both tumor and normal mucosa tissue demonstrated similar methylation levels across the genome, where at least two thirds of the 27 500 CpG sites showed a very low degree of methylation. The tumor and normal tissues showed very similar overall methylation patterns, however there was a slight net increase in the global DNA methylation levels in the tumor. This contrasts the belief that global DNA hypomethylation is a general hallmark of all cancer. In concordance with other recent studies we found increased DNA methylation in the tumors in the promoter regions of *TIMP3*, *SEMA3B*, *FBP2*, *CDH1*, *CDKN2A*, *MLH1*, and *SFRP1,2 and 5* [19–22, 26–31].

Hierarchical clustering of the entire dataset illustrated different methylation patterns between the tumor and the control tissue, and this exercise may distinguish tumor from adjacent tissue with relative accuracy. Statistically significant subclustering between the histological subtypes, according to the Lauren classification, was not seen, probably because of the low sample size. However, the aggregation of the intestinal tumor samples towards the right of the heat map in Fig. 3 may illustrate a separate methylation profile within this subtype, which could be more prominent in a larger study. Different DNA methylation profiles in the histological subtypes have been suggested by Wang et al. who demonstrated global hypomethylation intestinal type cancers, and gene promoter hypermethylation in diffuse type cancers [23]. This paradox may explain why several other authors in the past have reported both genome wide hypermethylation and hypomethylation in GC tissue.

### Correlation with clinicopathological parameters and gene expression

Further, we observed that DNA methylation of the *GFRA3* gene promoter showed significant correlation with almost all clinicopathological parameters. Most importantly, high methylation levels of *GFRA3* conferred a very unfavorable prognosis, with no high-expressing individuals surviving 20 months. *GFRA3* codes for the artemin receptor which mediates activation of the *RET* proto-oncogene, and has been implicated in a GC diagnostic and prognostic signature [24]. In breast cancer, increased expression of *GFRA3* was associated with lymph node metastasis and advanced tumor stage [32]. In pancreatic cancer *GFRA3* may be implicated in the promotion of the disease through increased cell motility and invasiveness [33, 34], and this gene is also up regulated in non-small cell lung cancer [35]. We expected to see down regulation of the *GFRA3* gene in the hypermethylated tumor specimens, however there was no significant association between the *GFRA3* methylation levels and gene expression in the study. Furthermore, it is widely accepted that there are distinct differences in the tumor biology between diffuse and intestinal GC. This difference, however, was not reflected in the *GFRA3* promoter methylation levels, as we found no differences in *GFRA3* methylation levels between the two histological types. *GFRA3* may play different roles in the two cancer types, however a larger study is necessary to clarify whether there exists a true difference between the methylation of this gene in the two histological types and its significance.

In our previous study [17], we found that *CLDN1* gene expression was highly associated with reduced post-operative survival, and that *IL-8* was the most highly upregulated gene in the tumor specimens. Although there was no association between the methylation levels of these genes and their expression, we wanted to investigate the relationship between the most prominent findings in our two studies: *CLDN1* and *IL-8* gene expression and *GFRA3* promotor methylation: Indeed, the expression of *CLDN1* was statistically associated with both the *IL-8* gene expression and *GFRA3* promotor methylation. The association between *GFRA3*, *IL-8* and *CLDN1* and the clinical features such as post-operative survival, lymph node metastasis, gastric metaplasia and patient age may describe a more complex relationship which we have not further evaluated in this study.

Between the 200 most differentially methylated CpG sites, and the 130 most differentially regulated genes, there was a significant inverse relationship between methylation and gene expression in 13 of the genes, confirming that DNA methylation may be one of several regulatory mechanisms of gene expression in GC. However, other mechanisms than DNA methylation must account for the majority of gene regulation, such as gene mutations, and other epigenetic mechanisms like histone modifications, nucleosome positioning, non-coding RNAs, and microRNAs.

The cause of hypermethylation in gastric cancer is unclear, nonetheless infectious agents may be an important contributing factor. There is a strong association between *H. pylori* and GC, recognized by the World Health Organization as a class 1 carcinogen [36]. *H. pylori* triggers the chronic inflammatory process that results in the mucosal transformation leading to GC, described by Correa [37] and later refined by Tahara [38]. The role of *H. pylori* in the methylation of gastric mucosal DNA, however, has not been extensively studied. It has been observed that *cag*^+^*H. pylori* infection results in both global hypomethylation in gastric mucosa [14–16] and hypermethylation of promoter regions of several tumor suppressor genes [39]. Chronic inflammation *per se* has also been shown to cause aberrant methylation in gastric epithelial cells [40]. Other infectious agents, such as the Epstein-Barr virus, have also been implicated in the promotion of DNA methylation in a subgroup of GC [39]. To evaluate the presence of *H. pylori* has not been the aim of this study. Up-regulation of the *IL-8* gene is a general inflammatory marker and is also associated with *H. pylori*-induced inflammation. The strong association between expression of *CLDN1* gene, methylation of *GFRA3* promoter and expression of *IL-8* gene in tumor tissue warrants further investigation in regards to the influence of *H. pylori* infection, but this is beyond the scope of this study.

Our main findings, such as the hypermethylated *GFRA3* gene promoter and its possible prognostic role, should be further studied in a larger cohort of patients in the future.

## Conclusion

In the present study, we demonstrated hypermethylation of the *GFRA3* promoter region in GC samples, and identified an inverse relationship between the degree of *GFRA3* hypermethylation and post-operative survival. *GFRA3* was also associated with *CLDN1* gene expression, a potential prognostic factor demonstrated in a previous study. The tumor and normal samples showed distinct DNA methylation profiles, indicating that aberrant DNA methylation may be a distinct feature of GC. Between the most aberrantly methylated gene promoters and corresponding gene expression there was only modest correlation, demonstrating that mechanisms other than DNA methylation must account for many of the changes in gene expression which occur in this disease.

## Additional file

Additional file 1: β values for selected CpG-sites. (TXT 7 kb)

## Abbreviations

GC: gastric cancer
CpG: cytosine-phosphate-guanine nucleotide sequence
CIMP: CpG island methylation phenotype
CLDN1: claudin-1
IL-8: interleukin-8
GFRA3: GDNF family receptor alpha 3
Cag: cytotoxin associated gene
PCR: polymerase chain reaction
KEGG: Kyoto Encyclopedia of Genes and Genomes
IF: impact factor
